# Retraction Note: New technique of end to side two layered and stented duct to mucosa pancreaticojejunostomy with omental wrapping during Whipple operation

**DOI:** 10.1186/s12893-026-04328-7

**Published:** 2026-11-09

**Authors:** Hesham A. Elmeligy, Ahmed M. Azzam, Yousra Ossama, Mahmoud Rady

**Affiliations:** 1 General Surgery Department, Theodor Bilharz Research Institute (TBRI), Giza, Egypt; 2 Environmental Research Department, Theodor Bilharz Research Institute (TBRI), Giza, Egypt; 3 Pathology Department, October 6 University, Giza, Egypt

**Retraction Note: BMC Surgery 25, 201 (2025)**

**https://doi.org/10.1186/s12893-025-02893-x**

The Editor has retracted this article. After publication, concerns were raised regarding the authenticity of the statistical data reported in Tables 1 and 2. The authors did not reply to repeated requests from the Publisher for clarification and source data. The Editor therefore no longer has confidence in the results and conclusions reported in this article.

The authors have not stated whether they agree or disagree with this retraction.

